# Using Routine Surveillance Data to Assess Dengue Virus Transmission Risk in Travelers Returning to the United States

**DOI:** 10.3201/eid3202.251217

**Published:** 2026-02

**Authors:** Kristyna Rysava, Zachary J. Madewell, Maile B. Thayer, Liliana Sánchez-González, Kamalich Muniz-Rodriguez, Ashley Brown, Julie Thwing, Laura E. Adams, Gabriela Paz-Bailey, Michael A. Johansson

**Affiliations:** University of Warwick, Warwick, UK (K. Rysava); Centers for Disease Control and Prevention, San Juan, Puerto Rico, USA (Z.J. Madewell, M.B. Thayer, L. Sánchez-González, K. Muniz-Rodriguez, L.E. Adams, G. Paz-Bailey, M.A. Johansson); Centers for Disease Control and Prevention, Atlanta, Georgia, USA (A. Brown, J. Thwing)

**Keywords:** Dengue virus, arboviral diseases, dengue surveillance, travel health notices, ArboNET, outbreak detection, emerging infectious diseases, traveler-based surveillance, viruses, mosquito-borne, vector-borne infections, United States

## Abstract

Dengue virus poses a growing global health threat, yet inconsistent local surveillance limits global risk assessments. We analyzed 10,530 travel-associated dengue cases among US travelers reported to ArboNET during January 2010–April 2024, involving travel to 128 countries. By using negative binomial and Poisson models, we developed country-specific thresholds (75th, 80th, 90th percentiles) to identify elevated travel-associated dengue risk. We applied a >10-case threshold in a 3-month period to improve specificity. The final dual-criteria method accurately identified high-risk periods, including sustained transmission in countries with limited official reporting, such as Cuba in 2022–2023. Threshold comparisons revealed a tradeoff between early detection and overclassification, whereas real-time and retrospective assessments revealed consistent high-risk signals. This traveler-based approach offers a timely, complementary method for travel-associated dengue risk detection, although timeliness might be reduced if reporting delays increase beyond our observations. Our findings support integrating travel surveillance into global dengue monitoring and preparedness efforts.

Dengue is a mosquitoborne viral disease of major public health concern, particularly in tropical and subtropical regions (*1*). Four dengue virus (DENV) serotypes, transmitted primarily by *Aedes* spp. mosquitoes, can cause repeat infections; secondary infections might be associated with more severe disease (*2*,*3*). An estimated 60%–80% of all DENV infections are asymptomatic or subclinical, complicating surveillance and control (*4*,*5*).

Dengue incidence has increased globally in recent decades, and nearly half the world’s population now lives in areas at risk (*6*–*8*). International travel further contributes to dengue burden, as infected travelers introduce dengue to new areas. Genomic and routine surveillance data indicate that DENV serotypes have moved repeatedly between regions, including introductions to and from the Caribbean that contributed to transmission across the Americas (*9*,*10*). Most evidence on viral spread comes from national surveillance systems, but travel-based surveillance data can provide complementary insights into DENV exposure risk, particularly where local data are limited or delayed, by detecting introductions or exportations through infected travelers. Traditional surveillance depends on timely case reporting, laboratory confirmation, and consistent national data sharing, all of which vary widely by country and can delay recognition of emerging outbreaks. Traveler-based surveillance, although influenced by who travels and where, can provide near–real-time indication of international transmission patterns that might otherwise go undetected. Integrating local and traveler-based surveillance streams can enhance detection of DENV exposure risk: local data reflect population-level risk, whereas traveler data capture exportation risk and can provide early warning where national reporting is incomplete. The concurrent emergence of other *Aedes*-borne viruses, such as chikungunya and Zika, has further highlighted the role of human mobility in arbovirus spread (*11*,*12*). Global dengue surveillance and the identification of high-transmission periods have become increasingly critical as international connectivity grows.

To increase awareness of global health risks, the US Centers for Disease Control and Prevention (CDC) issues Travel Health Notices (THNs) to advise travelers, clinicians, and public health professionals about disease outbreaks or other health concerns. THNs might be triggered by outbreaks, disease occurrence in new locations, or large events such as natural disasters or mass-gatherings and take into consideration both risk and the availability of preventive measures (*13*). Dengue THNs support both pretravel counseling and posttravel clinical evaluation: they inform travelers about destinations with elevated risk and help clinicians consider dengue in returned travelers with fever. That role has become more necessary with the recent rise in travel-associated dengue; 3,742 cases were reported in 2024, far exceeding the previous peak of 1,474 cases in 2019 (*14*,*15*). Historically, new THNs have relied on outbreak declarations or detailed national surveillance data to confirm that case numbers exceed historical levels. However, dengue surveillance varies widely by country, with differing testing practices, laboratory capacity, and public data availability, creating challenges for timely warnings.

To help address those gaps in surveillance, we developed a new approach to assess DENV incidence levels in other countries by using routine surveillance data from travelers returning to the United States. This approach leverages dengue cases reported to ArboNET, the national arboviral surveillance system, to establish baseline dengue activity in US travelers and detect deviations that indicate increased transmission (*16*). We used regression models and percentile-based thresholds, combined with a case-count criterion, to identify sustained periods of elevated risk by country and to support more timely, reproducible guidance for both pretravel risk communication and posttravel clinical decision-making.

## Methods

### Data Sources

For this study, we used dengue case data among travelers returning to the United States reported to ArboNET during January 2010–April 2024 (*16*). ArboNET is a national CDC surveillance system that monitors locally acquired and travel-associated arboviral diseases. Dengue is a nationally notifiable disease; state health departments receive reports from clinicians and laboratories, conduct case investigations, and submit data to CDC. ArboNET data are reviewed and updated continuously. Routine analyses and data pulls occur at least monthly to support surveillance and decision-making.

Countries of exposure are identified through public health investigations on the basis of travel history 14 days before symptom onset. For this analysis, we included all countries identified as exposure locations for >2 US travel-associated dengue cases during the study period. ArboNET includes the country of exposure, date of symptom onset, and US state of residence. All analyses were based solely on dengue cases in US travelers.

### Statistical Analysis

To identify elevated incidence periods in travelers, we fit country-specific regression models to monthly dengue case counts from January 2010–April 2024. Models included the month of symptom onset to capture seasonal variation and US state of residence to account for differences in reporting. We used negative binomial or Poisson regression, depending on the variance-to-mean ratio of case counts. For each country, we estimated the modeled distribution of expected monthly case counts and classified incidence as high (above the selected threshold), medium (between the median and the threshold), or low (below the median).

### Sensitivity of Risk Warning Thresholds Analysis

We evaluated percentile thresholds at the 75th, 80th, and 90th levels because those thresholds are commonly used in outbreak detection algorithms to balance sensitivity and specificity: lower percentiles provide earlier signals, whereas higher percentiles provide more conservative classifications (*17*). For each threshold, we quantified how often country-months were flagged as high risk and the duration of consecutive high-risk periods (mean, median, and maximum). Our analysis focused on 27 countries that reported >2 travel-associated dengue cases in 2014, the year with the median number of travel-associated cases across the study period, to ensure sufficient data for comparison. Those analyses evaluated the sensitivity of risk warnings to threshold selection, balancing timeliness and specificity.

### Real-Time Versus Retrospective Risk Assessments

To assess the effect of reporting delays on threshold classifications, we compared country-specific real-time case data (cases reported at the time of risk assessment) with complete retrospective data (full case data as of April 2024). For each country, we compared the duration of consecutive months with high-risk warnings under each percentile threshold in both datasets.

### Case Count Thresholds Assessment

Our objective was not to redefine CDC’s existing >10-case threshold for THNs but to embed it within a transparent, reproducible modeling framework by using percentile-based thresholds accounting for seasonal and historical variation. To refine alert criteria, we assessed a dual-criteria rule requiring both exceedance of the 80th percentile threshold and >10 travel-associated dengue cases in a 3-month window. The >10-case criterion reflects internal CDC THN practice and is a pragmatic cutoff for distinguishing sustained transmission from isolated events. We applied that dual rule across all country-months and compared the number and duration of alerts generated by the threshold-only versus dual-criteria approaches. 

### Final Alert Criteria for THNs

On the basis of those analyses, the final criteria we used to classify country-months as high dengue risk were exceeding the 80th percentile of the modeled distribution of historical travel-associated dengue case counts and reporting >10 travel-associated dengue cases in the prior 3 months (Appendix Figures 1, 2). This dual-criterion approach distinguishes sustained incidence from isolated spikes, especially in countries with low traveler volume. The 3-month window reflects ongoing elevation in dengue risk, accommodates reporting delays, and aligns with the monthly review cycle used for CDC THNs. We classified a country as high-risk for a given month if both criteria were met during any of the preceding 3 months. We completed all analyses by using R version 4.4.0 (The R Project for Statistical Computing, https://www.r-project.org).

## Results

### Dataset Overview

ArboNET recorded 10,530 travel-associated dengue cases during January 2010–April 2024, involving travel to 128 countries (Appendix Table 1). During 2010–2023, the overall median delay between symptom onset and reporting date was 1.3 months (interquartile range [IQR] 0.7–2.7) (Appendix Figure 3), ranging from 1.9 months (IQR 1.1–3.9) during 2010–2019 to 2.8 months (IQR 1.3–10.3) in 2020–2021 and 1.3 months (IQR 0.9–2.2) in 2022–2023. Real-time data missed a median of 25% (IQR 15%–39%) of retrospective high-risk months across countries, with country-specific proportions ranging from 0% (e.g., Barbados, Jamaica, Peru) to 100% (Kenya) (Appendix Table 2).

### Temporal Patterns

**Figure 1:**
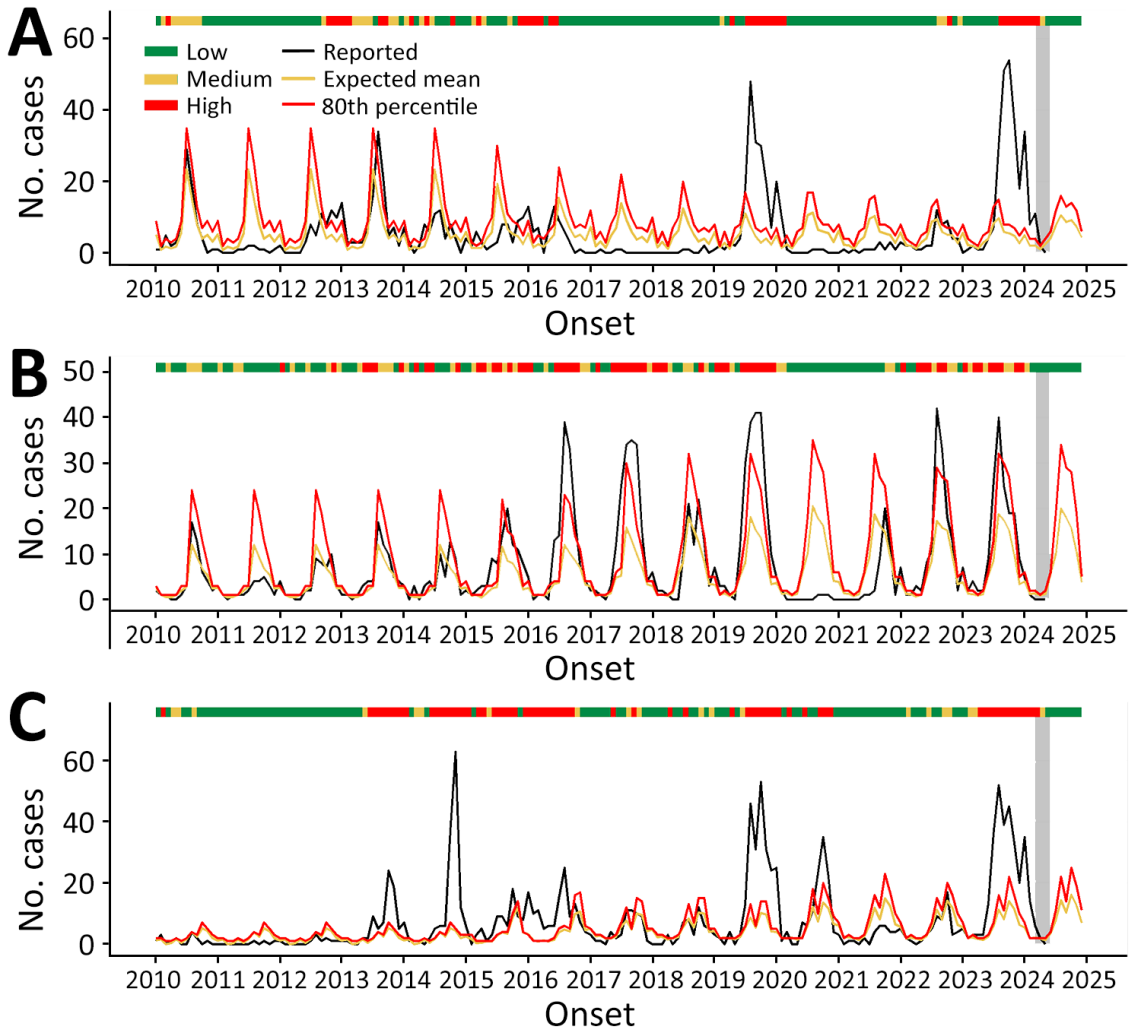
Temporal patterns and modeled transmission classification levels on the basis of percentile thresholds for dengue cases in travelers returning to the United States from the Dominican Republic (A), India (B), and Mexico (C), 2010–2024. Black line represents monthly dengue cases reported in US travelers. Yellow line represents expected mean number of cases on the basis of the fitted model. Red line represents the 80th percentile of the fitted distribution. Risk levels were categorized as low (green bar), medium (yellow bar), or high (red bar). We made those classifications on the basis of modeled case counts and did not incorporate the >10-case count criterion used in the Travel Health Notice classification. The most recent months of reported data at the time of analysis are shaded in gray to indicate periods of incomplete reporting. All 3 countries showed strong seasonal patterns and multiple sustained periods where traveler case counts exceeded the 80th percentile, reflected in frequent high-risk classifications, particularly during recent years (2022–2024).

We observed clear seasonal fluctuations in dengue cases among US travelers, stratified by country, which were reflected in the estimated thresholds. By using an 80th percentile threshold derived from retrospective models, we found that some countries experienced sustained high risk over multiple years, whereas others had more sporadic elevations (Figure 1; Appendix Figures 4, 5). Costa Rica (58 months), Haiti (57 months), Mexico (44 months), Dominican Republic (41 months), and the Philippines (41 months) had the most months retrospectively classified as high-risk, indicating frequent and prolonged traveler-associated incidence. Cuba demonstrated a more recent pattern, with 36 high-risk months from late 2018 to early 2024, including the highest number of traveler cases in a single month, 241 cases in August 2022. India (40 months), Thailand (37 months), Jamaica (36 months), Brazil (31 months), and Honduras (30 months) also demonstrated persistent incidence. In contrast, some countries had more sporadic dengue incidence with fewer high-risk months, such as French Polynesia (13 months), Ecuador (5 months), Peru (5 months), and Sri Lanka (5 months), reflecting short-lived increases in cases among travelers.

### Sensitivity of Risk Warning Thresholds and the Duration of Risk Warnings

**Figure 2:**
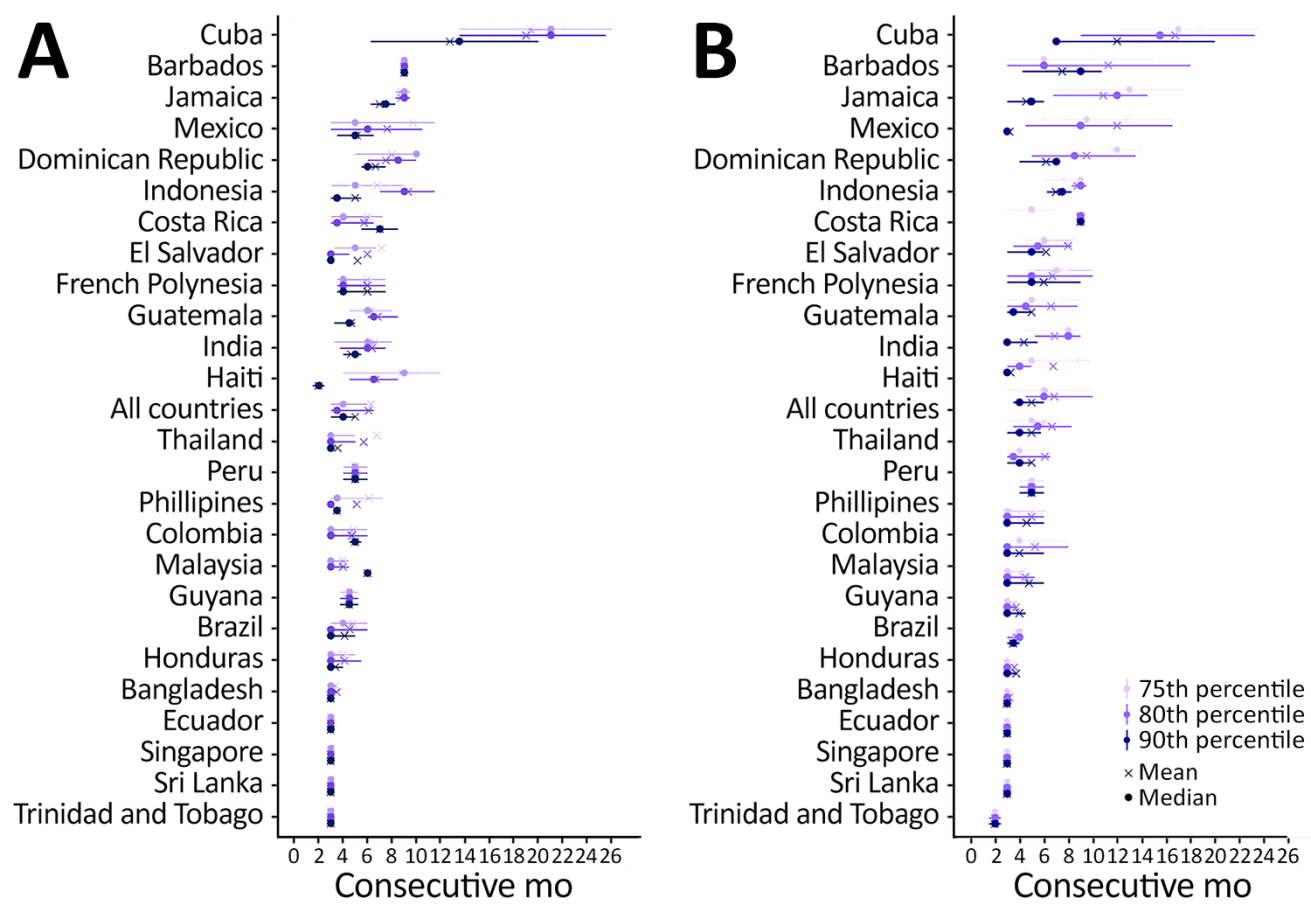
Duration of dengue outbreak warnings by country of exposure for travelers returning to the United States, based on different percentile thresholds in retrospective and real-time datasets, 2010–2024. A) Retrospective (complete) ArboNET data. B) Real-time data available at each monthly timepoint. Panels show the distribution of consecutive months classified as high-risk for dengue based solely on the 75th, 80th, and 90th percentile thresholds. Horizontal lines represent the interquartile range of outbreak duration for each country; median and mean warning lengths are indicated.

We found that by applying the threshold-only approach, the frequency of high-risk months increased with lower percentile thresholds (Figure 2; Appendix Figure 6). Some countries, such as India and the Philippines, had substantially fewer high-risk months at the 90th percentile than at the 75th percentile, whereas countries including Guatemala and Jamaica showed more stable patterns and were less sensitive to threshold choice.

The median duration of elevated-risk warnings, which we defined as consecutive months classified as high risk on the basis of threshold exceedance alone, was 5 months at the 80th percentile (Appendix Figure 6). At the 90th percentile, the median warning duration was 3 months, whereas at the 75th percentile it also reached 5 months. Cuba had the longest warning durations, a median of 15.5 months and a maximum of 30 months at the 80th percentile, reflecting prolonged high-risk periods. India (median 12 months) and Mexico (median 6 months) also showed extended warnings. Sri Lanka, Malaysia, and Ecuador had consistently short warning durations (3–4 months), indicating more transient activity or lower traveler volume.

Threshold choice also influenced the overall frequency of high-risk months. Across all countries, we classified a median of 33% (IQR 11%–46%) of months as high risk at the 75th percentile, compared with 29% (IQR 11%–42%) at the 80th percentile and 19% (IQR 9%–25%) at the 90th percentile (Appendix Table 3), demonstrating that stricter cutoffs reduced the proportion of months flagged as elevated risk.

### Assessment of Risk Levels with Real-time Data

When we compared real-time and retrospective data, we found the average number of months that dengue cases crossed the threshold was similar (Figure 2; Appendix Figure 6). However, differences emerged by country and threshold level. Honduras, Guatemala, and Jamaica showed little change in the number of high-risk months when using real-time data versus retrospective data. In contrast, fewer months were flagged by using real-time (compared with retrospective) data for Brazil, Dominican Republic, Haiti, India, and the Philippines, particularly at the 80th and 90th percentiles, indicating reduced sensitivity to sustained transmission when relying only on contemporaneous reports. Some countries, including Barbados and Colombia, had high-risk periods identified only in the retrospective assessment, not detected in real-time, across all 3 thresholds, underscoring the effect of reporting delays and gaps (Appendix Figure 6).

Although the number of months crossing the threshold was generally consistent, the mean duration of outbreak warnings on the basis of real-time data was shorter than for retrospective data (Figures 2; Appendix Figure 6). For instance, at the 80th percentile threshold, the median warning duration was 3 months for real-time data (IQR 3.0–6.0), compared with 4.5 months for retrospective data (IQR 3.0–7.0) among countries with >2 dengue cases in 2014. The narrower IQR in real-time data suggests that incomplete reporting can interrupt otherwise continuous outbreaks, effectively chopping longer episodes into shorter ones and reducing sensitivity to sustained transmission, especially where healthcare-seeking and reporting practices vary.

### Addition of Case Count Thresholds

**Figure 3:**
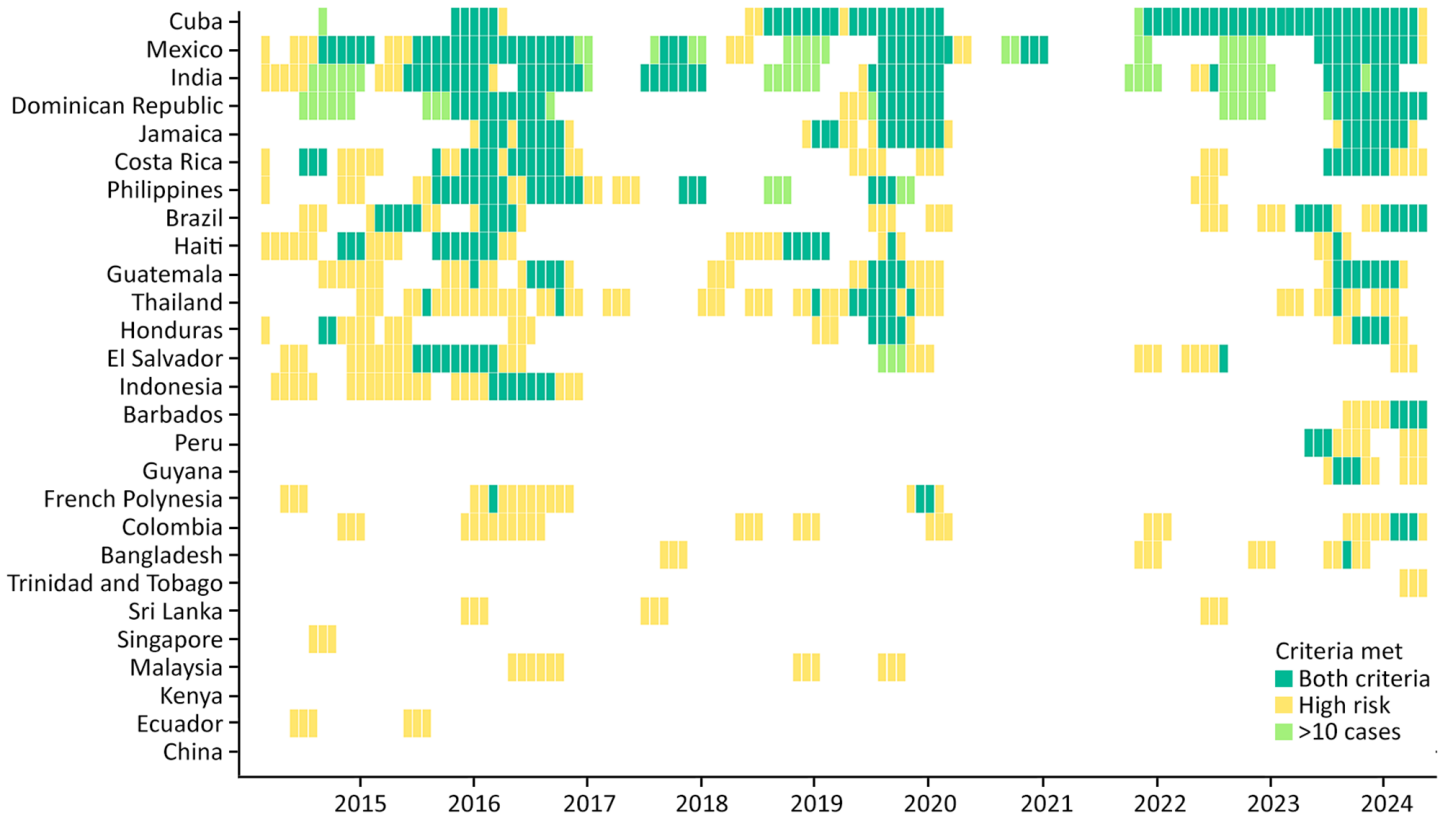
Monthly dengue risk classifications among travelers returning to the United States, by country, by using real-time data and dual-alert criteria, 2014–2024. Each tile represents 1 country-month and is colored according to the criteria met: dark green for months that met both criteria (dengue case counts exceeded the 80th percentile threshold and met the >10 case-criterion in the previous 3-month window), yellow for months exceeding the 80th percentile threshold only, and light green for months meeting the >10-case criterion but below the transmission threshold. Blank tiles indicate months that met neither criterion. The high transmission threshold was based on the 80th percentile of modeled country-specific traveler case distributions (real-time or annually updated). Countries are ordered by the total number of months that met both criteria.

**Figure 4:**
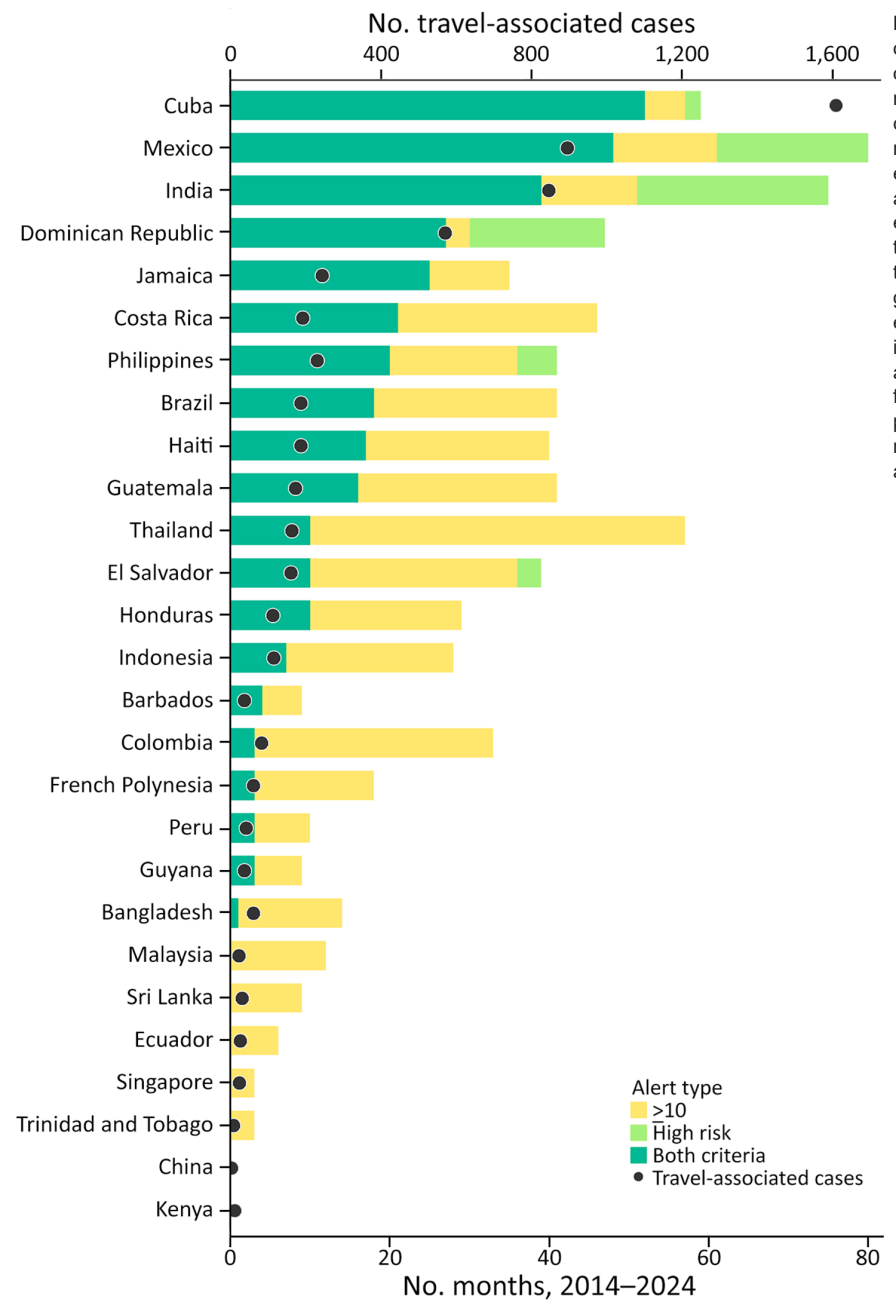
Travel-associated dengue case volume and number of alert months among travelers returning to the United States, by country, 2014–2024. Stacked bars represent the number of months each country was classified as meeting both criteria (teal), exceeding the 80th percentile threshold only (yellow), or meeting the ≥10-case criterion only (light green); categories are mutually exclusive, as in Figure 3. Dots indicate the total number of travel-associated dengue cases reported from each country over the same period. The secondary y-axis reflects case counts scaled to align with the alert-month axis.

Applying the dual-criteria rule, which required both threshold exceedance and >10 cases in a 3-month period, reduced the number of THN alerts by 52.8% from 714 to 337. The magnitude of reduction varied by country (Figures 3, 4). In higher-volume origins such as Cuba and Mexico, most high-risk months also met the ≥10-case criterion, whereas in others, such as Thailand, Colombia, and Malaysia, many threshold-exceedance months did not meet the case-count criterion.

To contextualize alert frequency by country, we also visualized total travel-associated case volume alongside alert months (Figure 4). Countries with higher dual-criteria alert frequencies generally correspond with higher traveler case volumes. For example, Cuba reported 1,609 travel-associated dengue cases and met both criteria in 52 months, whereas countries such as Singapore, Kenya, and China had both low case counts and few or no alert months.

### Final Criteria and Application to Travel Health Notices

We applied the final dual-criteria alert approach to identify country-months with sustained dengue incidence relevant to US travelers beginning in 2024. We then compared monthly traveler-based alerts to historical CDC THNs. The new method identified substantially more high-risk periods than THNs issued during the same interval (Appendix Figure 7). Although THNs often captured isolated or short-lived signals, typically 1- or 2-month spans, the new criteria consistently flagged longer durations of elevated incidence. From mid-2023 to early 2024, Brazil, Costa Rica, Cuba, Dominican Republic, Guatemala, Jamaica, and Mexico all experienced extended multi-month periods flagged by the new method, whereas corresponding THNs were limited or absent. For example, no THNs were issued for Brazil or Costa Rica despite 6–10 consecutive months meeting both incidence and case-volume criteria. Some official outbreak notifications during the study period were not captured by the final alert criteria (Appendix Figure 7), reflecting the inherent limitations of relying solely on traveler-based data.

## Discussion

This study demonstrates that national surveillance for travel-associated dengue can detect periods of heightened dengue activity and provide early warning of increased incidence in international destinations. We identified seasonal patterns by country and derived country-specific thresholds that account for expected trends. We also detected high dengue incidence in countries with limited public reporting, such as Cuba, where sustained activity was evident from late 2018 to early 2024, highlighting the added value of traveler-based surveillance. Outbreak detection varied by percentile threshold, with the 80th percentile providing a useful balance between sensitivity and specificity. When we compared real-time with retrospective data there were generally consistent elevated-risk detections, although reporting delays shortened the apparent duration of warnings for some countries. When we applied a minimum case count threshold (>10 cases in 3 months) we noted an increased specificity while maintaining sensitivity, minimizing low-volume anomalies and consistently identifying prolonged high-risk periods aligned with known outbreaks.

Changes in dengue incidence patterns inferred from traveler data depend on factors including the number of travelers, travel routes, and individual prevention measures, which are difficult to quantify. Because ArboNET did not capture the total number of US travelers to each country, we could not calculate incidence rates or directly adjust for traveler volume. Instead, we analyzed trends in absolute case counts by using thresholds that incorporate historical and seasonal patterns, providing a practical and interpretable approach in the absence of denominator data. Traveler-based data also do not necessarily mirror local transmission intensity. Countries with inadequate reporting systems might appear to have few outbreaks in official statistics while traveler data reveal ongoing transmission; conversely, concentrated tourism in low-risk areas might yield lower traveler risk than national averages suggest. Those discrepancies underscore both the value and limitations of using travelers as a proxy for local populations. Because of those limitations, this framework is intended as guidance for US travelers and health practitioners and as a complement, not a replacement, to in-country surveillance.

Our findings indicate that traveler cases can reliably capture seasonal trends and detect increases above expected levels. Seasonal patterns aligned with known dengue transmission seasons across multiple countries, and stronger signals were found in destinations frequently visited by US travelers, including the Caribbean, Mexico, and Central America. Fewer cases were detected from more distant dengue-endemic countries, such as Singapore and Kenya, likely reflecting lower US traveler volume. India was an exception of note, with clear seasonal trends and high-incidence months consistently detected across 14 years, likely reflecting substantial travel between the United States and India (*18*). Although a single traveler population will not provide equal indicators of risk for all countries, traveler-based thresholds remain highly relevant for informing US THNs and could be adapted by other countries by using their own traveler surveillance data.

Our results support using traveler-based dengue surveillance as one component of a broader decision-making framework for timely risk communication through THNs. The 2023 spike in cases among travelers from Costa Rica, Dominican Republic, Honduras, Jamaica, and Mexico, all surpassing the 80th percentile, highlights the need for timely, country-specific THNs. In contrast, some destinations did not reach high-risk thresholds, either because of better-controlled transmission or lower traveler volume, enabling more targeted responses rather than uniform interventions. Operationally, traveler-based surveillance could be used as a routine supplement to THN reviews, flagging destinations that exceed traveler-based thresholds even when local reporting is incomplete. Integration into dashboards or automated alerting systems would enable CDC and partner agencies to cross-check traveler signals against official reports, strengthening risk communication for travelers and clinicians.

Despite improved specificity and sensitivity, the final alert criteria did not capture several official outbreak notifications, underscoring a crucial limitation of traveler-based surveillance. Traveler data should not be used alone to infer local incidence; integrating multiple surveillance sources remains essential for comprehensive global dengue risk monitoring. In countries with sparse traveler data, alerts could be improved by combining traveler-based signals with regional context, historical travel volume, or syndromic surveillance from other sources. Linking traveler thresholds with airline passenger data or mobility datasets might also strengthen interpretability when case numbers are low.

Although traveler-based surveillance offers advantages in sensitivity and timeliness, particularly for sustained transmission that might be underreported locally, it is not intended to replace existing systems. Official publications and outbreak alerts remain critical for identifying emerging risks, as in-country surveillance, when available, provides the most reliable indicator of disease trends. Traveler-based data can nonetheless serve as an early warning system where official dengue reporting is incomplete or delayed (*9*,*19*,*20*). This approach aligns with the World Health Organization’s International Health Regulations, which emphasize early detection and rapid response to public health threats of international concern (*21*,*22*), and illustrates how model-based approaches can be integrated with traditional surveillance to support more complete risk assessment.

A key insight from our study is that real-time and retrospective data produced similar outbreak warnings, suggesting the traveler-based threshold approach is robust to moderate reporting delays. Further improvements in real-time data collection and reporting, through better coordination among clinicians, laboratories, and public health agencies at state and federal levels, would still enhance the timeliness of outbreak alerts and public health responses (*17*,*23*).

The first limitation of our study is that dengue case detection in travelers depends on healthcare-seeking behavior and diagnostic practices in the United States, and many infections are likely underreported; we assume underreporting is relatively consistent over time, enabling reliable trend analysis. Second, traveler data might not fully reflect transmission in origin-country populations because findings depend on where travelers go and how travel volume changes, including in response to mass gatherings or other events. Without data on total US travelers by destination, we cannot calculate incidence rates or separate changes in transmission from changes in travel volume. Although such events could drive short-term increases, our dual-criteria approach reduces the effect of transient spikes by focusing on sustained incidence and still provides useful information for travelers visiting elevated-risk locations. Third, thresholds are based on reported case counts, which are subject to underreporting and variable healthcare-seeking behavior in both origin countries and the United States. If surveillance systems capture only 10%–30% of symptomatic dengue infections, then true incidence is substantially higher than observed, but thresholds should still capture relative deviations from baseline, which is the primary goal for outbreak detection. Fourth, reporting delays in ArboNET, median 1.3 months during 2010–2023, limit real-time utility because some cases are not available until weeks after onset, and reporting lag remains a constraint on timeliness. Fifth, the approach may be less sensitive in destinations infrequently visited by US travelers, limiting generalizability, although it could be adapted by other countries using their own traveler surveillance. Finally, transmission dynamics evolve over time, and growing global dengue incidence will require periodic updates of warning thresholds, with more frequent updates during major epidemics or when real-time decision-making is needed.

In conclusion, travel-associated dengue surveillance provides a practical, timely complement to national systems, particularly for travelers visiting high or emerging dengue-risk regions. Our dual-criteria method offers a transparent framework that can be adapted by other countries by using traveler or sentinel surveillance data. Future work should validate thresholds against independent datasets, explore integration with mobility and genomic data, and assess cost-effectiveness for real-time implementation. Embedding traveler-based analyses into existing decision-making frameworks, such as CDC’s THN process and the World Health Organization’s International Health Regulations, could strengthen global preparedness and support more timely, targeted responses to dengue outbreaks.

Appendix Additional information about using routine surveillance data to assess dengue virus transmission risk in travelers.
