# Supplementary material for: Using Routine Surveillance Data to Assess Dengue Virus Transmission Risk in Travelers Returning to the United States
Source: Emerg Infect Dis. 2026 Feb;32(2):202–10. doi: 10.3201/eid3202.251217 (PMC12928239; doi:10.3201/eid3202.251217)
Supplement: Appendix — Additional information about using routine surveillance data to assess dengue virus transmission risk in travelers. [file 25-1217-Techapp-s1.pdf]

Article DOI: https://doi.org/10.3201/eid3202.251217

EID cannot ensure accessibility for supplementary materials supplied by authors. Readers who have difficulty accessing supplementary content should contact the authors for assistance.

Using Routine Surveillance Data to Assess Dengue Virus Transmission Risk in Travelers Returning to the United States

Appendix

Appendix Table 1. Annual travel-associated dengue case counts among U.S. travelers, by country of exposure, 2014–2024. Values represent the total number of laboratory-confirmed dengue cases reported to ArboNET for each country and year. Countries are listed alphabetically. These counts provide the underlying data for model-based thresholds and risk classifications shown in Appendix Figure 3.

Country	2014	2015	2016	2017	2018	2019	2020	2021	2022	2023	2024
Bangladesh	3	11	6	7	8	16	0	9	12	18	1
Barbados	1	1	3	1	0	0	3	1	0	7	28
Brazil	6	43	29	3	1	23	4	3	26	38	60
China	3	1	0	0	1	1	0	0	0	0	0
Colombia	8	8	17	2	8	8	5	7	5	15	16
Costa Rica	35	34	55	2	2	17	4	2	13	84	10
Cuba	23	37	11	11	66	281	9	26	930	370	42
Dominican Republic	55	59	65	3	4	167	34	13	50	206	55
Ecuador	4	7	5	5	0	0	0	6	4	4	1
El Salvador	19	83	18	3	9	29	4	9	31	6	2
French Polynesia	14	6	21	5	7	17	7	0	0	0	0
Guatemala	15	17	28	8	11	38	3	4	13	86	5
Guyana	3	1	1	1	0	1	4	1	4	30	2
Haiti	47	58	23	5	62	27	4	0	0	19	0
Honduras	24	9	11	0	1	44	1	6	7	25	5
India	50	93	135	167	91	210	10	45	141	164	6
Indonesia	18	25	48	10	10	19	4	4	13	10	3
Jamaica	4	9	65	6	12	80	15	2	2	64	13
Kenya	3	1	2	1	1	4	2	2	0	3	1
Malaysia	2	5	6	4	2	5	3	0	1	4	0
Mexico	133	77	128	56	46	210	138	31	58	254	57
Peru	3	1	3	0	1	0	0	1	3	29	9
Philippines	24	34	68	40	41	54	10	2	23	14	5
Singapore	4	3	5	1	1	5	2	1	4	3	0
Sri Lanka	3	5	5	12	2	5	1	1	6	4	1
Thailand	13	30	28	22	33	51	1	0	5	24	3
Trinidad and Tobago	2	2	5	0	0	0	0	0	0	0	2

Appendix Table 2. Proportion of retrospective high-risk months missed in real-time assessments, by country, 2010–2024. Values represent the number of country-months classified as high risk using retrospective data but not identified as high risk using real-time data (missed months), divided by the total number of retrospective high-risk months for that country. Proportions illustrate the impact of reporting delays on outbreak detection in real-time assessments.

Country	Retrospective high-risk months	Missed months (real-time)	% missed
Kenya	9	9	100.0
Singapore	9	6	66.7
Sri Lanka	19	10	52.6
Ecuador	12	6	50.0
India	87	36	41.4
Philippines	61	25	41.0
Guyana	15	6	40.0
Dominican Republic	47	17	36.2
French Polynesia	27	9	33.3
Malaysia	18	6	33.3
Indonesia	40	12	30.0
Bangladesh	19	5	26.3
Brazil	55	14	25.5
El Salvador	48	12	25.0
Trinidad and Tobago	4	1	25.0
Mexico	79	18	22.8
Honduras	37	8	21.6
Costa Rica	57	11	19.3
Haiti	48	8	16.7
Thailand	66	10	15.2
Cuba	67	10	14.9
Guatemala	48	7	14.6
Colombia	35	2	5.7
Barbados	9	0	0.0
Jamaica	35	0	0.0
Peru	10	0	0.0
China	0	0	--

Appendix Table 3. Proportion of country-months classified as high risk under different percentile thresholds, 2010–2024. Values represent the percentage of total country-months flagged as high-risk dengue periods using the 75th, 80th, and 90th percentile thresholds in retrospective analyses. Across all countries, the median proportion of high-risk months declined as thresholds became stricter: 33% (IQR: 11–46%) at the 75th percentile, 29% (IQR: 11–42%) at the 80th percentile, and 19% (IQR: 9–25%) at the 90th percentile. These results highlight how threshold selection influences the sensitivity of outbreak classification.

Country	% months high-risk (75th)	% months high-risk (80th)	% months high-risk (90th)
Bangladesh	15.4	15.4	11.4
Barbados	8.1	7.3	7.3
Brazil	45.5	44.7	28.5
China	0.0	0.0	0.0
Colombia	33.3	28.5	18.7
Costa Rica	48.0	46.3	25.2
Cuba	61.0	54.5	48.8
Dominican Republic	42.3	38.2	24.4
Ecuador	9.8	9.8	9.8
El Salvador	41.5	39.0	25.2
French Polynesia	24.4	22.0	19.5
Guatemala	45.5	39.0	28.5
Guyana	12.2	12.2	9.8
Haiti	41.5	39.0	13.0
Honduras	34.1	30.1	16.3
India	74.0	70.7	26.0
Indonesia	35.0	32.5	24.4
Jamaica	30.9	28.5	22.8
Kenya	7.3	7.3	7.3
Malaysia	14.6	14.6	12.2
Mexico	74.8	64.2	36.6
Peru	8.1	8.1	8.1
Philippines	56.9	49.6	18.7
Singapore	7.3	7.3	7.3
Sri Lanka	17.9	15.4	4.9
Thailand	57.7	53.7	32.5
Trinidad and Tobago	3.3	3.3	3.3

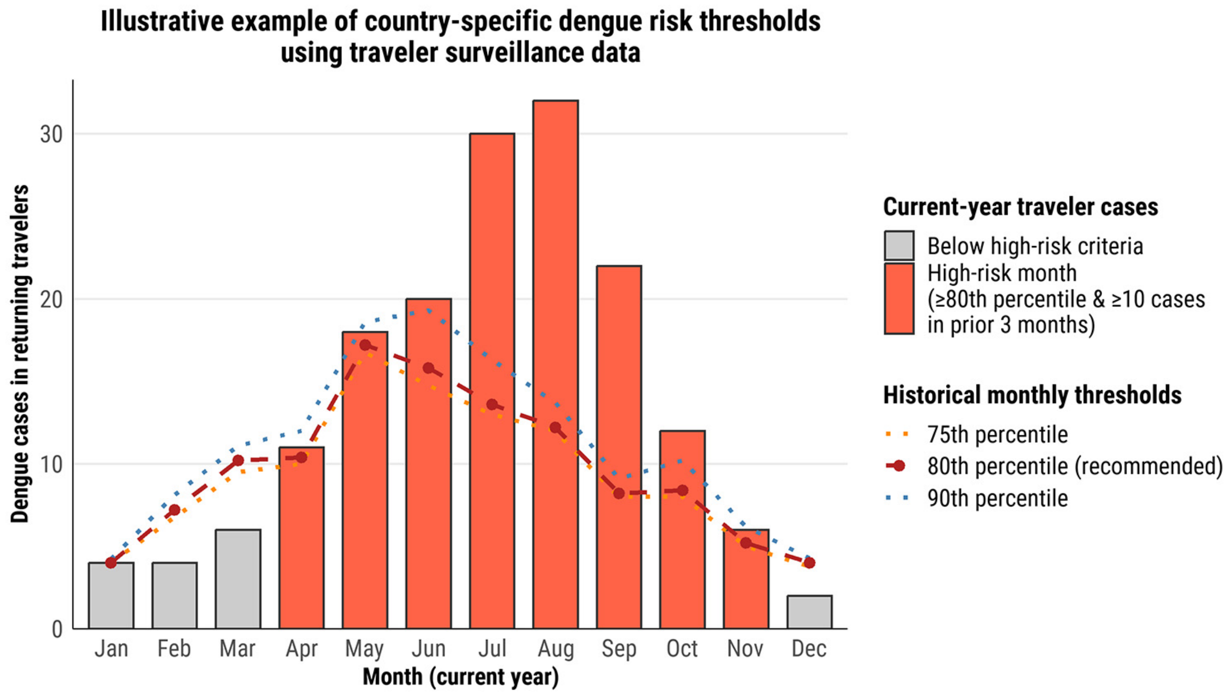

Appendix Figure 1. Illustrative example of country-specific dengue risk thresholds using traveler surveillance data. Bars show monthly dengue cases among returning travelers for a hypothetical destination (current year). The dashed red line indicates the historical 80th percentile threshold in traveler cases for each month, derived from the preceding 10 years of data; dotted lines show 75th and 90th percentile thresholds. Red bars indicate months meeting the recommended high-risk criteria: cases ≥80th percentile and ≥10 cases in the previous 3 months. Data are simulated for demonstration and do not represent any specific country.

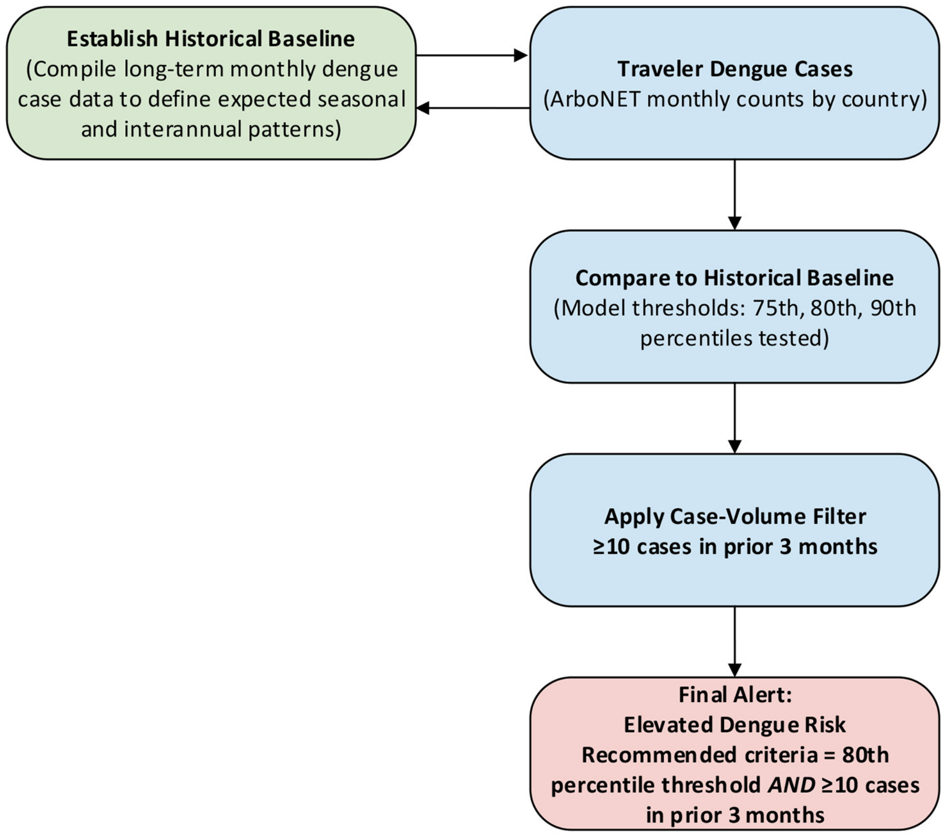

Appendix Figure 2. Framework for identifying elevated dengue risk in international destinations using traveler surveillance data. Historical baselines are established from long-term dengue case data to model expected seasonal and interannual patterns. Monthly traveler case counts from ArboNET are compared to these baselines to detect threshold exceedance, filtered by case volume (≥10 cases in the prior 3 months). Elevated dengue risk is defined as exceeding the 80th percentile threshold and reporting ≥10 cases within the preceding 3 months.

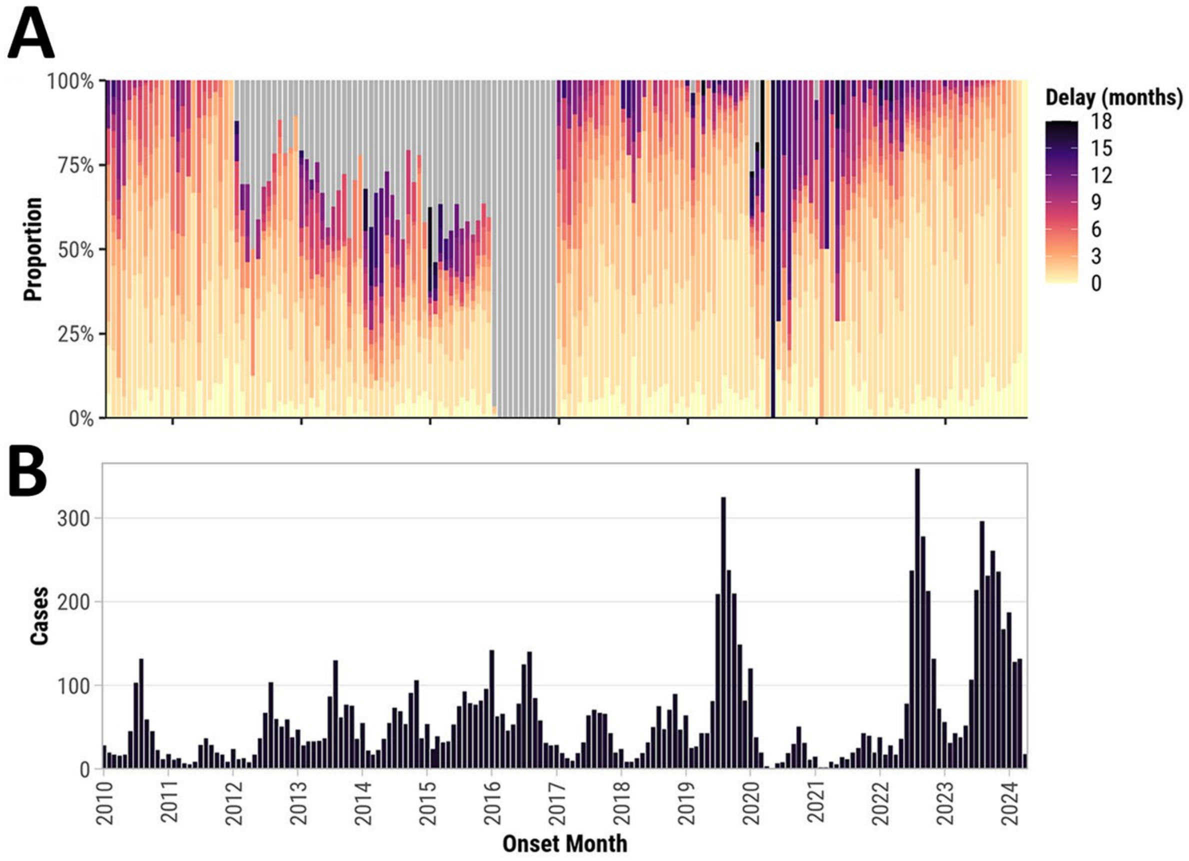

Appendix Figure 3. Monthly reporting delays for travel-associated dengue cases in ArboNET, 2010–2024. (A) The proportion of cases reported each month by delay in months between symptom onset and reporting. A continuous color gradient is used: light yellow indicates no delay, with progressively darker colors representing longer delays (up to 18 months). Gray bars indicate missing onset or report dates. This panel shows that the timeliness of case reporting varied substantially over time. Delays were shortest during 2022–2023, but prolonged from 2020–2021, coinciding with the COVID-19 pandemic. A gap from mid-2016 to early 2017 reflects missing report dates for many cases during this period, which prevented calculation of reporting delays. (B) The absolute number of dengue cases by onset month, showing seasonal and interannual variation in traveler-associated case counts. Peaks in 2019, 2020, and 2022 correspond to periods of elevated transmission in endemic regions. Together, these panels highlight how reporting delays fluctuate over time and may impact the ability to detect and respond to emerging dengue trends in near real-time.

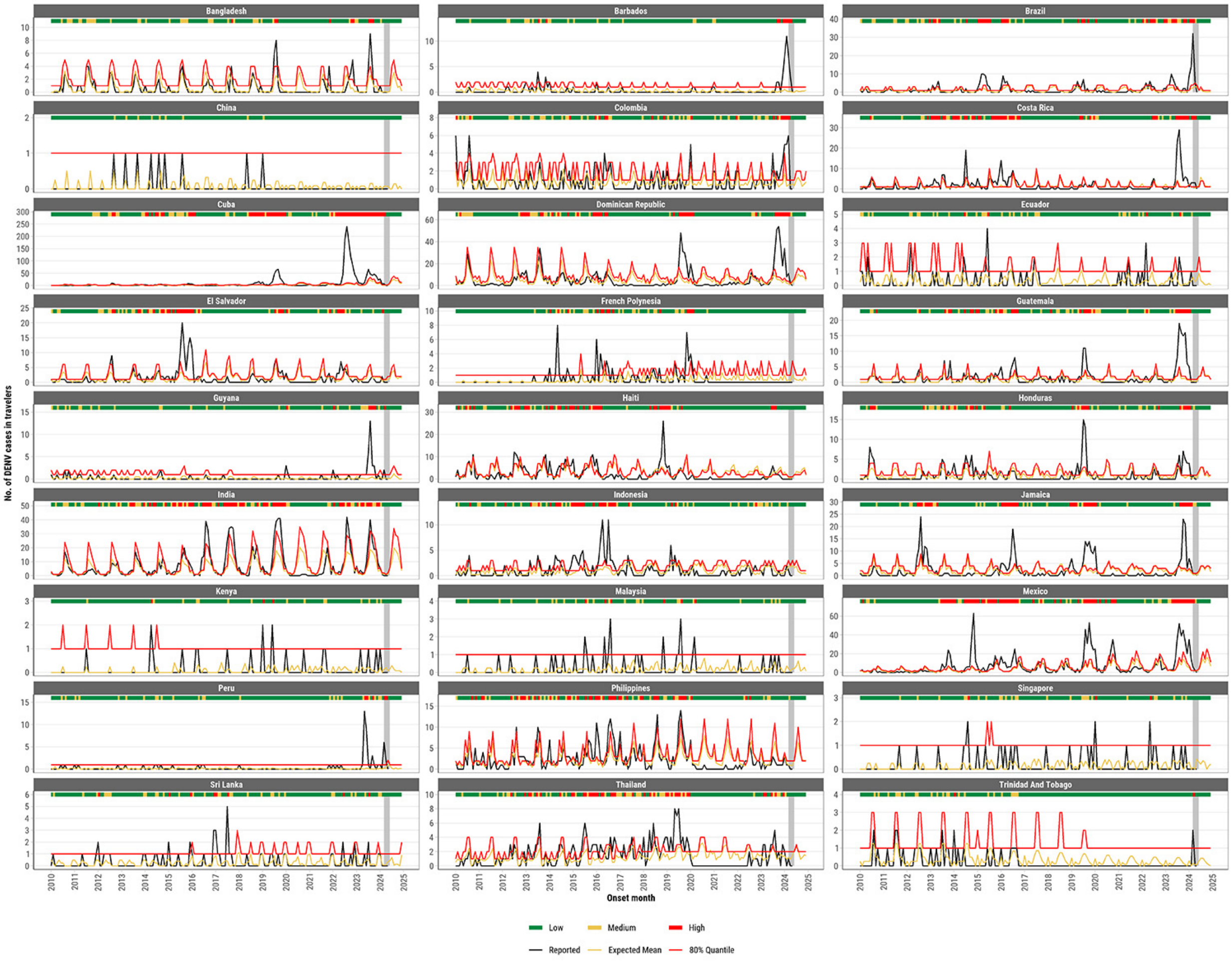

Appendix Figure 4. Temporal dengue case trends and modeled monthly transmission classification levels for U.S. travelers from 33 countries, 2010–2024. The time series plot illustrates monthly reported dengue cases among U.S. travelers (black line), expected mean number of cases based on the fitted model (yellow line), and the 80th percentile of the fitted distribution (red line). Risk levels were categorized as low, medium, or high based on these thresholds and are indicated by green, yellow, and red shaded bars at the top of each panel, respectively. Thresholds were updated annually using only data available up to each year, simulating a real-time surveillance approach. The most recent months of reported data at the time of analysis are shaded in gray to indicate periods of incomplete reporting. Countries shown had more than two travel-associated dengue cases reported in 2014—a year chosen as representative of median dengue activity across the dataset—to ensure broad geographic inclusion for descriptive visualization. This differs from the ≥10-case threshold applied in later analyses, which was used to reduce false-positive alerts during real-time risk classification. Many countries show recurring seasonal spikes in dengue activity (e.g., Brazil, Philippines, India), while others show more sporadic or localized peaks (e.g., El Salvador, Sri Lanka). The figure highlights variability in dengue risk across countries and demonstrates how model-based thresholds captured both persistent and short-term traveler-associated transmission patterns.

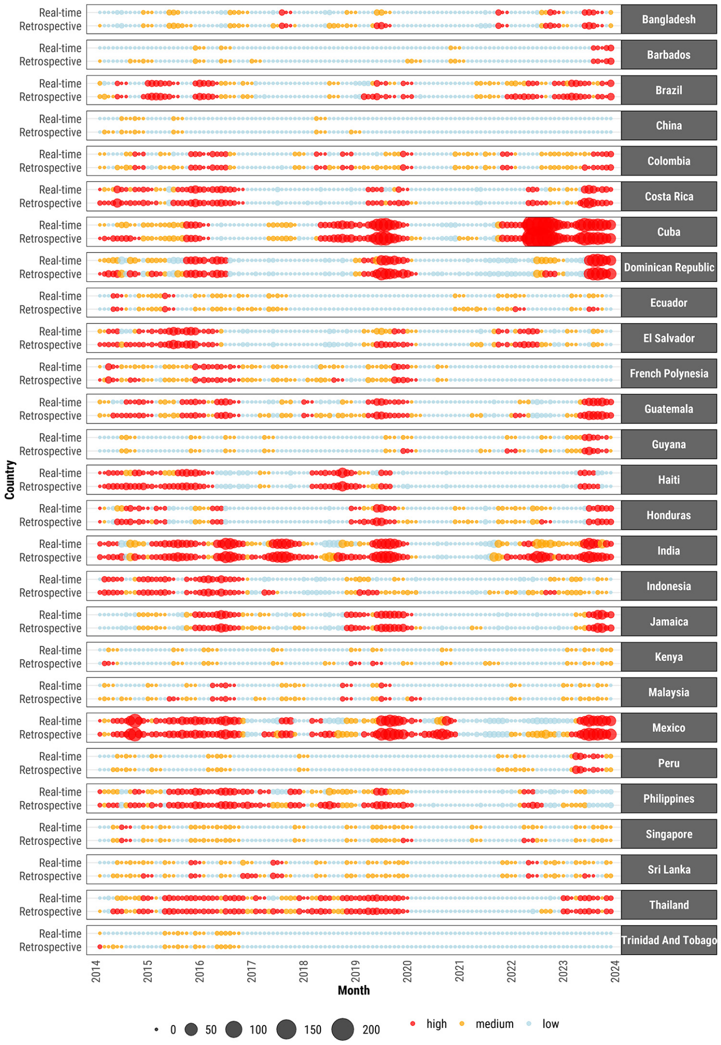

Appendix Figure 5. Dengue case counts and risk classifications among travelers by country and month, using real-time and retrospective data, 2014–2024. Each bubble represents the number of travel-associated dengue cases reported from a specific country and month; bubble size corresponds to case count, and color reflects model-derived dengue risk level using an 80th percentile threshold. Risk levels were classified as high (red), medium (orange), or low (blue) based on whether observed monthly case counts exceeded the 80th percentile, were between the median and 80th percentile, or were below the median of modeled historical values. The top panel shows classifications using real-time data (based only on data available at each time point), while the bottom panel uses retrospective data (based on complete data from 2010–2024). This figure illustrates the impact of reporting delays on risk classification. For example, India and Mexico show more months classified as high risk in retrospective data, reflecting cases that were reported after the month of interest. Conversely, countries like Guatemala and Jamaica show high concordance between real-time and retrospective assessments. Overall, the bubble plots highlight seasonal and geographic variation in dengue activity among travelers and underscore the added value of retrospective data for identifying sustained high-risk periods.

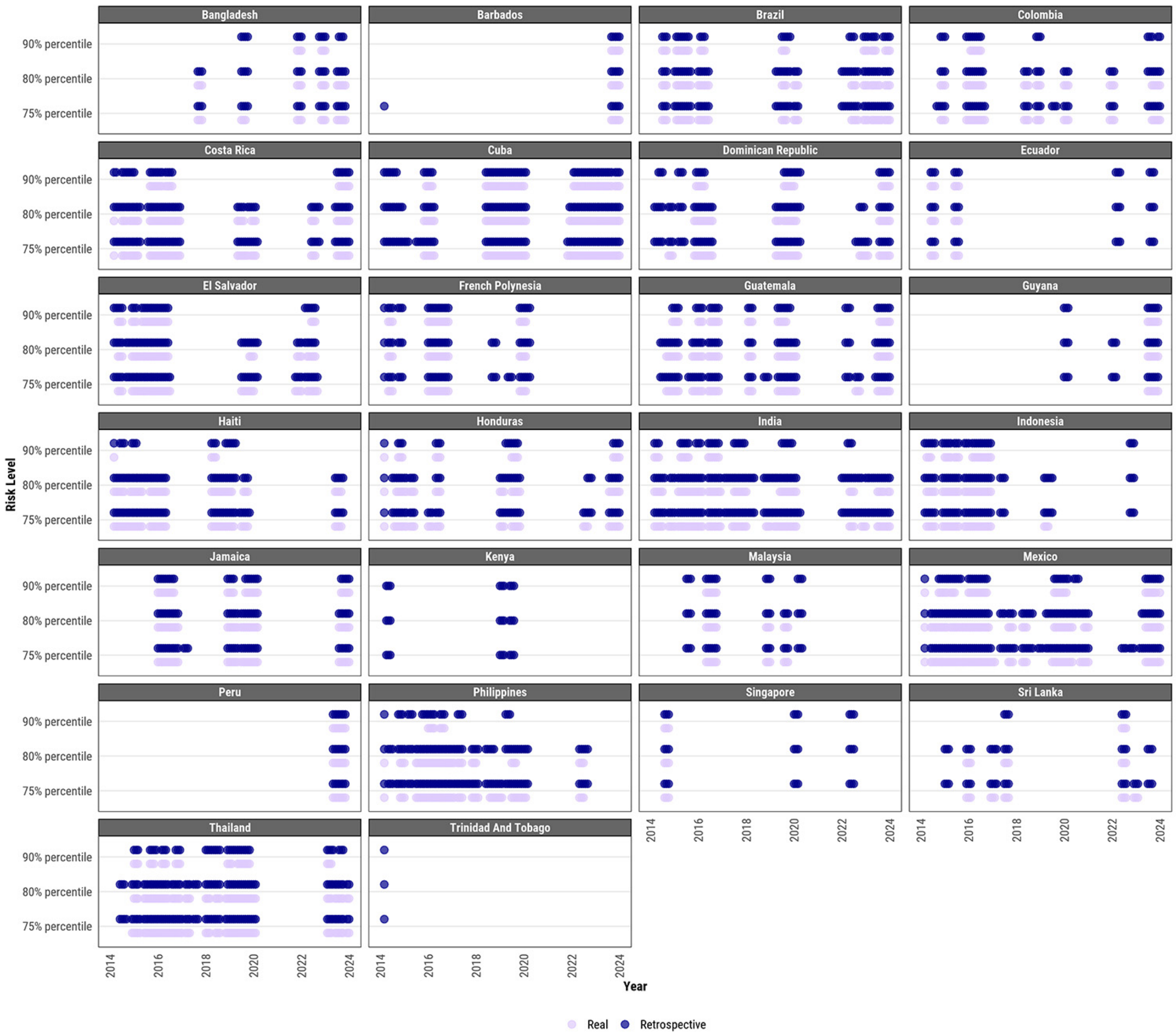

Appendix Figure 6. Comparison of real-time and retrospective traveler-based dengue outbreak warnings using 75th, 80th, and 90th percentile thresholds, 2014–2024. Each point represents a country-month in which travel-associated dengue case counts exceeded a specified percentile threshold (75th, 80th, or 90th) based on modeled historical data. Rows correspond to percentile thresholds and colors indicate whether the high-risk month was detected using real-time data (i.e., case data and thresholds available up to that month) or retrospective data (i.e., using complete case data through April 2024). For example, in India, both real-time and retrospective methods captured frequent warnings at the 80% and 75% thresholds, but some months—especially at the 90% threshold—were only detected retrospectively, likely due to delays or incomplete reporting at the time of assessment. This comparison illustrates how sensitivity to outbreak detection varies by threshold and highlights the impact of reporting lags: retrospective data often detect more warnings, especially at higher thresholds, underscoring the tradeoff between timeliness and completeness in real-time risk surveillance.

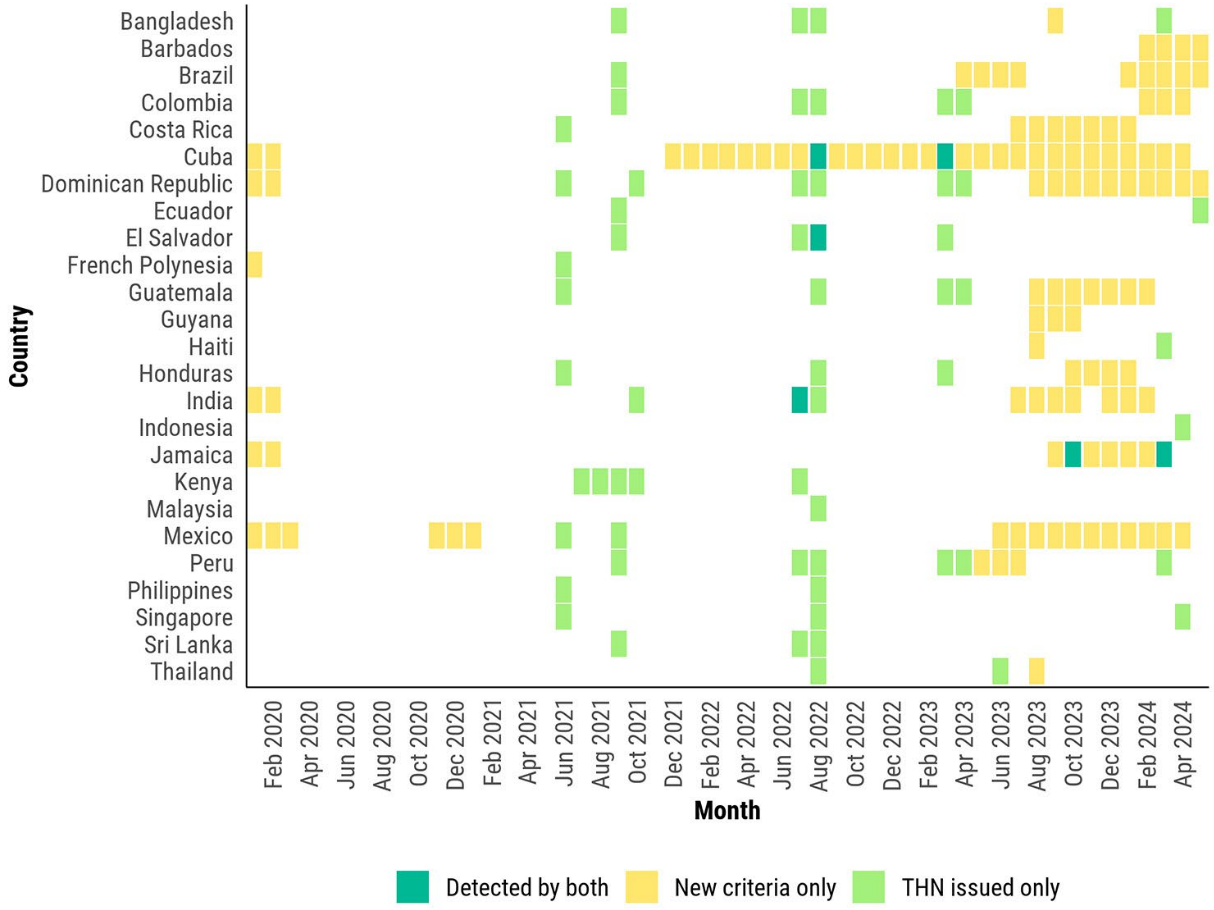

Appendix Figure 7. Comparison of monthly dengue risk classifications using traveler-based criteria vs. CDC Travel Health Notices (THNs), 2020–2024. Each tile represents a country-month and is colored by detection method: yellow indicates high-risk periods flagged only by the new traveler-based criteria (≥10 cases in 3 months and exceeding the 80th percentile threshold), light green indicates months with official THNs but not flagged by the new method, and dark green denotes agreement between both approaches. This comparison highlights the greater sensitivity of traveler-based surveillance in detecting sustained dengue activity. For example, countries such as Brazil, Costa Rica, Cuba, and Mexico showed long stretches of high transmission identified by the new method (yellow or dark green), often without corresponding THNs. In contrast, some short-term signals detected by THNs (light green) were not captured by the traveler-based method, particularly in countries with low traveler volume. These differences underscore the value of complementary data streams: the traveler-based method enhances outbreak detection for persistent transmission patterns, while THNs may reflect official notifications or context-specific concerns not evident in traveler data alone. Note: This comparison is limited to 27 countries that reported ≥2 travel-associated dengue cases in 2014, selected to ensure reliable model fitting and consistent evaluation across countries. Many countries that received official THNs during this period were not included in this subset and are not shown here.
